# Response: “Commentary: Mental distress in patients with cerebral visual injury assessed with the German Brief Symptom Inventory”

**DOI:** 10.3389/fnagi.2015.00158

**Published:** 2015-08-17

**Authors:** Carolin Gall, Doreen Brösel, Gabriele H. Franke

**Affiliations:** ^1^Department of Rehabilitation Psychology, AHW, Magdeburg-Stendal University of Applied SciencesStendal, Germany; ^2^Department of Psychiatry, AMEOS HospitalHaldensleben, Germany

**Keywords:** mental health, quality of life, cerebral visual injury, coping with disease, mental distress, visual field defects

In his valuable commentary on our recent publication Ashley Craig pointed out that the observed “strong connection between mental distress and a disorder like cerebral visual injury is highly important for directing future research in the area.” Within this context we wish to emphasize another significant factor: patients' quality of life that may be relevant as a potential mediator variable.

Besides generic health-related quality of life, vision-related quality of life as assessed with questionnaires such as the National Eye Institute—Visual Functioning Questionnaire (NEI-VFQ, Mangione et al., 2001) are important factors that may help to explain why some patients develop mental health impacts after cerebral injury while others do not. The NEI-VFQ may be used to gather information on how patients with visual field loss after lesions to the visual pathway are able to *cope* with daily visual tasks. For brain-damaged patients the NEI-VFQ may be ideally conducted together with a neuroophthalmological supplement which is available in English and German (Raphael et al., 2006; Wagenbreth et al., 2011).

Recently, we have observed that a higher extent of vision-related quality of life in patients with cerebral visual injury was related to lower levels of mental distress (Gall et al., 2013). We further hypothesized that the maintenance respectively elevation of vision-related quality of life could reduce and prevent mental distress due to vision problems. Thus, patients with persisting visual field defects may benefit from rehabilitation aiming at the development of individualized coping strategies. With a greater repertoire of coping strategies for visual demands in everyday life vision-related quality of life is likely to increase which may eventually translate into improved mental health.

Dr. Craig also highlighted the other side of the hypothesis, i.e., that “quality of life will improve should mental health problems be addressed” (Gall et al., 2015). In fact, it is very probable that the respective interventions—of which too few are being made available to visually impaired patients in need—may have a positive influence on both mental health and quality of life.

As pointed out in the commentary, very similar phenomena of elevated mental distress have been observed in patients with physical disorders after damage to the central nervous system (Craig, 2015). A next step forward may be to go beyond a cross-sectional methodology in order to study the occurrence of mental health problems with an economic questionnaire approach in longitudinal studies, for instance using the *Brief Symptom Inventory* (BSI, Franke, 2000), together with disease-related quality of life instruments, e.g., in post-stroke patients who likely suffer from sensory and physical symptoms at the same time. For the patients' benefit these studies should comprise an interventional approach targeting either neurological rehabilitation including designated coping strategies and/or psychotherapeutic interventions.

## Conflict of interest statement

The authors declare that the research was conducted in the absence of any commercial or financial relationships that could be construed as a potential conflict of interest.
